# Four-Dimensional Printing for Hydrogel: Theoretical Concept, 4D Materials, Shape-Morphing Way, and Future Perspectives

**DOI:** 10.3390/polym13213858

**Published:** 2021-11-08

**Authors:** Syed Sarim Imam, Afzal Hussain, Mohammad A. Altamimi, Sultan Alshehri

**Affiliations:** Department of Pharmaceutics, College of Pharmacy, King Saud University, Riyadh 11451, Saudi Arabia; maltamimi@ksu.edu.sa (M.A.A.); salshehri1@ksu.edu.sa (S.A.)

**Keywords:** 4D-printing technique, 4D-printed hydrogel, 4D shape morphing, comparative analysis, challenges and future perspective

## Abstract

The limitations and challenges possessed in static 3D materials necessitated a new era of 4D shape-morphing constructs for wide applications in diverse fields of science. Shape-morphing behavior of 3D constructs over time is 4D design. Four-dimensional printing technology overcomes the static nature of 3D, improves substantial mechanical strength, and instills versatility and clinical and nonclinical functionality under set environmental conditions (physiological and artificial). Four-dimensional printing of hydrogel-forming materials possesses remarkable properties compared to other printing techniques and has emerged as the most established technique for drug delivery, disease diagnosis, tissue engineering, and biomedical application using shape-morphing materials (natural, synthetic, semisynthetic, and functionalized) in response to single or multiple stimuli. In this article, we addressed a fundamental concept of 4D-printing evolution, 4D printing of hydrogel, shape-morphing way, classification, and future challenges. Moreover, the study compiled a comparative analysis of 4D techniques, 4D products, and mechanical perspectives for their functionality and shape-morphing dynamics. Eventually, despite several advantages of 4D technology over 3D technique in hydrogel fabrication, there are still various challenges to address with using current advanced and sophisticated technology for rapid, safe, biocompatible, and clinical transformation from small-scale laboratory (lab-to-bed translation) to commercial scale.

## 1. Introduction

Recently, several advanced and soft materials have been developed with diverse functionalities for 3D printing/bioprinting to fabricate complex designs using a smart technique. However, most of them resulted in dead printed objects or restricted their utility whenever time-evolving shape transformation was required. In 21st century, radical development and advancement in the diverse domains of science and technology takes place. Four-dimensional printing technology emerged as result of a significant transition in existing 3D printing and conventional manufacturing processes. The concept of 4D printing was first introduced and termed by Tibbit Skylar (MIT scientist, director of the self-assembly laboratory in 2013) as requiring stimuli response, dependent on time, predictably self-evolving, and made with dynamic material or construct (capable of transforming its shape over time) [1]. Notably, 4D printing is an innovative technology and imparts new dimensions in transformation over time in response to external stimuli, such as (a) physical (thermal, electrical, magnetic, UV and visible light, and ultrasound), (b) chemical (pH, water, and organic), and (c) biological responses (biomolecules) [2]. The advanced 4D materials (shape-morphing material or additive manufacturing material) can be programmed to adapt the dynamic behavior of set parameters of environment and subsequent transformation (reversible, irreversible, and semireversible) of their shape over time. Therefore, this technology is progressively considered to alleviate scientists’, researchers’, and programmer’s concerns to implement in a wide range of biomedical and engineering fields [3]. Recently, several additives of manufacturing of smart materials (time-dependent sensational behavior) have been explored by exposure to distinct external stimulations for diverse clinical and nonclinical applications in active research, thus overcoming numerous challenges. Four-dimensional printing technology is considered advantageous over 3D printing and overcomes several challenges associated with 3D printing. Fundamentally, there are three basic requirements (transformation variations) to materialize a 4D-printing process, which are (a) the preparation of stimuli-responsive composite material (3D material), (b) a specific stimulus to trigger a particular response in the environment, and (c) the length of time for shape transformation and response outcome [4]. The shape-shifting phenomenon (from 1D to 1D, 1D to 2D, 2D to 3D, and 3D to 4D over time) in a structure may be self-folding, self-twisting/bending, surface curling, linear/nonlinear expansion or contraction, reversible or irreversible transformation, and/or generation of surficial topographical features over time under specific stimuli [5]. This idea was originated using a hydrophilic material immerged in water to be activated [5].

The capability to create a 3D hydrogel would enable a range of clinical applications in tissue engineering (tissue or organ repair, biosensors, biomedicines, and biomedical uses) and 3D bioprinting for structured hydrogel in the macromolecular field [6]. However, 3D-printed hydrogel is often limited by ink insufficiency (lack of printable ink), challenged viscosity, and mechanical strength. Moreover, the current 3D approaches of hydrogel generation are associated with several severe challenges for printing cantilevered, hollow tubular structures, including weak mechanical strength in the hydrogel and poor cell viability for bioink (bioprinting) [7]. Therefore, 4D-printing technology emerged to solve these conventional issues of 3D printing and can be further shaped to develop dynamic devices for desired functionalities on demand over time [8]. To date, there are three types of materials to realize 4D printing, including (a) shape-memory polymers (SMPs), (b) hydrogels, and (c) other extracted biomaterials possessing shape-memory effect (SME) [9]. SMP-based 4D printing offers structural modifications/recoveries in response to stimuli (thermal), and such printing may inspire the molecular architecture of shape-memory hydrogels (SMHs). Hydrogels are a readily synthesized material with distinct advantages over synthetic polymeric materials. Hydrogels can be characterized as having high water content, biocompatibility, low cost, tunable toughness, and sustainability in wet environments, whereas synthetic SMPs have several challenges, such as lack of sustainability in wet environments, rigidity, permeability, stiffness, lipophilicity, and biological incompatibility [10]. Thus, SMPs cannot completely substitute hydrophilic soft materials due to ascribed limitations. It is noteworthy that self-morphing and mechanically active hydrogels are capable of undergoing desired programmable 3D-shape transformation and exhibiting mechanical tasks, as soft constructs (materials) under applied triggers have recently gained the interest of various scientists. Four dimensionally based hydrogels can be used to fabricate promising candidates for biomedical applications, such as targeted therapy, noninvasive diagnosis, cell manipulation, and implant placements [11,12,13]. Biomimetic 4D-printing hydrogel was realized using direct ink print and subsequent actuation employing an anisotropic dynamic transformation behavior in water (aqueous solvent-based stimuli) [14]. In this review, we summarized findings of 4D-printing technology to generate hydrogel and polymeric nanocomposites for biomedical, clinical, and nonclinical applications. 

## 2. Dimension (Scale) and Response Time of 4D-Printed Hydrogels

Notably, the dimensions of hydrogel-based, 4D-printed devices, structures, and constructs are still in millimeter scale, and the shape transformation under certain external stimuli takes several minutes. However, an electrostatically modified, anisotropic 4D-printed hydrogel actuator under rapid thermal stimuli (thermoresponsive actuation enabled by permittivity switching) (1/10th of second) has also been realized using cofacially oriented electrolyte nanosheets [15]. Authors reported that the distance between the nanosheets contracted and expanded under cooling and heating, respectively, in the absence of significant water uptake and release [15]. In practice, the development and fabrication of such structures at the microscale that possess prompt response speed are still crucial for targeted drug delivery, biomedical application, and tissue engineering. A thermoresponsive, microscale hydrogel with a reconfigurable helical structure has been constructed, and the response time was enhanced by increasing the heating rate [16]. In a further advancement, femtosecond laser (two photon enabled) direct printing has been constructed with features of nanoscale resolution, ultralow thermal impact, and excellent geometry to create 3D hydrogel microstructures for promising biomedical and photonic applications [17]. Microscale 4D-printed, hydrogel-fabricated constructs with quick swelling ability have also been realized. Kaehr and Shear fabricated a multiphoton chemically responsive protein hydrogel for microactuation where scanning nonlinear excitation was used to cross-link proteins at submicrometer 3D coordinates [18]. Furthermore, proteins differing in hydration properties can be combined to obtain a tunable (rapid and reversible) volume change (˂1 s) in response to variations in chemical environments [18]. The 4D printing of hydrogel with high architectural complexity and multiple freedoms of shape morphing still remains to be further explored. 

## 3. Comparative Analysis of 3D Hydrogel Printing, 4D Hydrogel Printing, and SMP-Based 4D Printing

In recent years, several advanced and soft materials have been explored for 3D printing/bioprinting to design complex structures using smart technique. However, various restrictions associated with 3D printing limited clinical, biomedical, and bioengineering applications. The ability of the shape-morphing feature of smart materials (or stimuli-responsive materials) over time under certain physical, chemical, and biological stimuli in 4D-printing technology gained the interest of scientists and researchers working in diverse biomedical fields (bioengineering, biosensors, actuators, tissue engineering, diagnosis, and therapeutic). SMPs are a class of shape-memory polymeric materials that can fabricate programmed complex designs under external stimuli over time. These materials retain two, or sometimes three, shapes as final structures (triple SMPs) after exposure to a stimulus [19]. A comparative analysis of 3D-printing hydrogel, 4D-printing hydrogel, and 4D-printing SMPs is summarized in Table 1.

## 4. Stimuli and Shape Transformation in 4D Printing for Hydrogel

**Figure 1:**
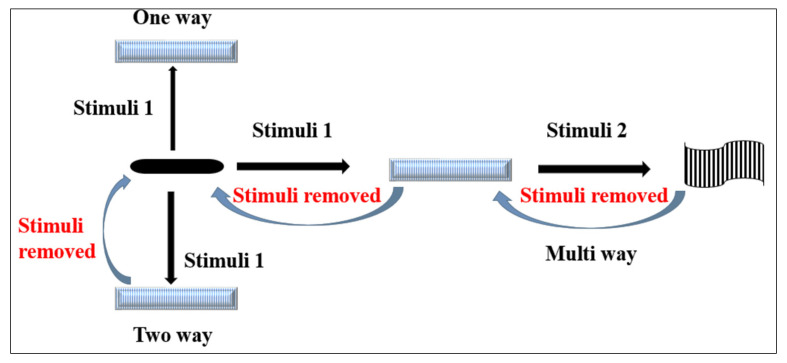
Schematic illustration of various shape transformations (one way, two way, and multiway) in response to stimuli.

**Figure 2:**
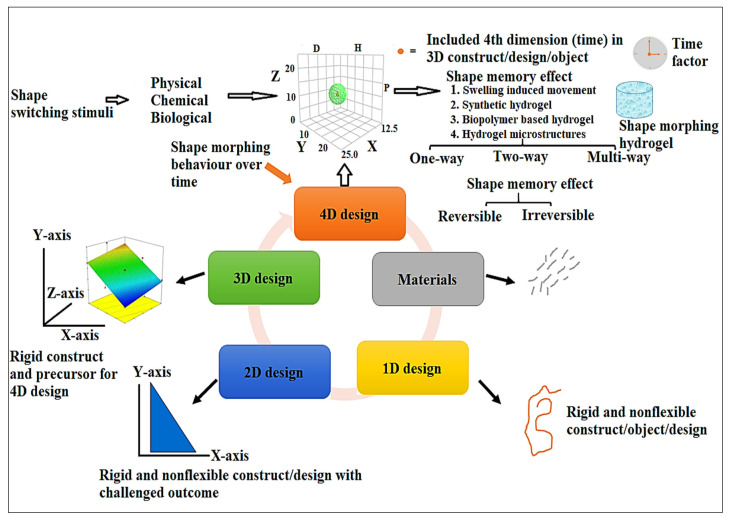
The evolutionary concept of 1D transformation to 4D transformation in hydrogel. Four dimensional design hydrogel under several stimuli (shape-switching stimuli) to achieve temporary reversible/irreversible shape-memory effect (SME).

Generally, various literature has been published regarding types and nature of shape transformation in response to exposed stimuli. These can be broadly classified as (a) physical, (b) chemical, and (c) biological stimuli [2]. Physical stimuli-based, smart shape-morphing materials are magnetic nanoparticles (magnetic-stimulus responsive), conducting polymers or nanoparticles (electrically responsive), photosensitive (chemophore groups) nanomaterials (light responsive), amphiphilic materials (thermoresponsive), and ultrasound-sensitive materials (sound-responsive polymers, such as biocompatible polycaprolactone for 4D bioprinting). Chemical stimuli-based, shape-morphing materials are pH responsive acidic/basic polymers (pH responsive) and hydrophilic functional groups with cross-links (moisture or solvent responsive). Finally, biologically active ligand molecules, which are probe linked with polymer or nanomaterial for targeted functionalization under physiological conditions, are biological stimuli-responsive biomolecules [2]. These advanced, smart shape-morphing, and stimuli-responsive materials execute irreversible (one way), reversible (two way), and multiway (observed in triple shape-morphing materials) deformations as illustrated in Figure 1 and Figure 2. Plasticity or deformability is commonly observed in polymer when exposed to chemoresponsive stimuli such as pH, water, and aqueous solvents for swelling purposes in 4D printing of hydrogel [20]. Hydrogel as comprised of hydrophilic, shape-morphing materials (gelatin, carbopol, alginate, hyaluronic acid, and methacrylated forms of these polymers) is capable of expanding throughout its volume by aqueous solvent, resulting in distinct functionalities upon exposure to altered aqueous environments for biomedical applications (biosensor, processors, actuator, bioprinting, and drug release) [21]. These smart hydrogels undergo a sol-gel transition (physically cross-linked hydrogel with netpoint formation or cleavage as the typical basis for this transition) and a swelling/shrinking shape transformation (covalently cross-linked hydrogel as the basis for this transition), which results in broad attention from scientists. Notably, a swelling/shrinking transformation of covalently cross-linked hydrogel is substantially induced by alterations in pH, temperature, ion concentration, polarity, hydrophobicity, functional group, nanoparticle addition, and charge inclusion of hydrophilic polymer [22]. As illustrated in Figure 1, shape switching as a one-way transformation (under stimuli 1 responsive effect) resulted in dual shape effects (from a temporary shape to a permanent shape) to construct designs using various individual polymeric components for permanent netpoints. For a thermally induced, shape-morphing hydrogel in polymer, glass transition temperature (*T_g_*), fusion temperature, melting point of polymer, and liquid-crystalline phase transition serve as molecular switches [23]. Moreover, triple-shape polymer or multishape polymer for multiway transformation can be generated by forming and recalling various types of temporary cross-links, such as a combination of PCL (polycaprolactone) and PEG (polyethylene glycol) or PCL and poly(cyclohexyl methacrylate) [24]. Interestingly, perfluorosulfonic acid ionomer exhibits dual-, triple-, and quadruple-shape effects with broad thermal transitions and without change in composition [25]. In case of multiple (multiway) reversible transformation (actuation in hydrogel) without intermediate programming, two-way shape switching is required. This was demonstrated for a poly(cyclooctene) cross-linked network using crystallization-induced elongation (CLE) and melting-induced contraction (MIC) [26]. Temperature-sensitive SMPs possessing hydrophilic chain segments on exposure to water can activate switching of those materials exhibiting water-induced plasticization or solvation as observed in swelling of poly(vinyl alcohol) networking in an aqueous system [27].

## 5. Four Dimensional Dynamic and Shape-Morphing Way 

**Figure 3:**
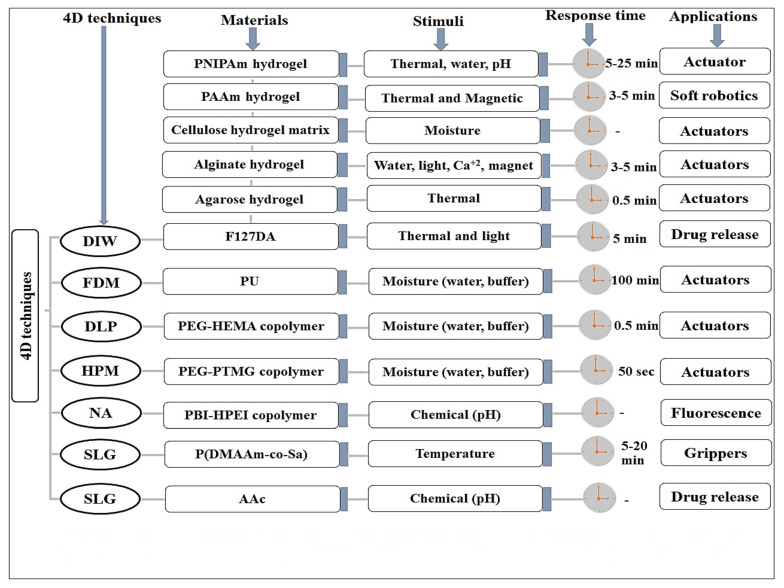
Illustration of various 4D hydrogel fabrication techniques, SMHs, stimuli, response time, and biomedical application (use). DIW: Direct ink writing, FDM: Fusion deposition modeling, ME: Microextrusion, SLG: Stereolithography, DLP: Digital light processing, NA: Not applied, HPM: Hot processing modeling [110].

**Figure 4:**
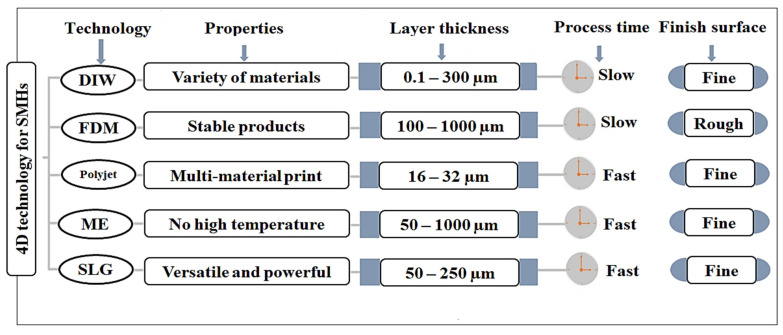
Illustration of various 4D hydrogel fabrication techniques, surface properties, process time, and layer thickness of 4D constructs. DIW: Direct ink writing, FDM: Fusion deposition modeling, ME: Microextrusion, SLG: Stereolithography [111].

Despite possessing several physicochemical and biologically compatible properties of the materials used in fabrication, these materials respond to certain specific ways of transformation under directed stimuli. This is illustrated in Figure 1, where reversible and irreversible shapes by one-way, two-way, and three-way (multiway) shape morphing were exhibited. In the literature, various 4D dynamic materials were reported for biomedical application, actuation, surgery, and drug delivery, which are classified and described in following subsections (Figure 3 and Figure 4).

### 5.1. One-Way Shape Morphing 

There were few 4D materials reported to execute one-way shape morphing in response to thermal stimuli. These are copolyester thermoplastic elastomer (TPC), poly(lactide) (PLA as polylactic acid)-HA (hyaluronic acid) copolymer, polycaprolactone triol, multi-isocyanate, and castor oil implemented to fabricate valve stent, orthopedic implant, and biomedical scaffold, respectively. Moreover, these constructs were designed using the FDM (fused deposition modeling) technique for one-way shape transformation [28,29,30]. Generally, shape-morphing materials (polymers) are initially modified/functionalized by adding other particular components to make them printable and introduce their specific functionality. For instance, benzyl methacrylate (a linear chain builder) was used to synthesize a self-healing, shape-memory polymer by adding poly(ethylene glycol) dimethacrylate as a cross-linker and polycaprolactone (PCL) as a self-healing agent to develop a 4D-printing construct [31]. Injectable thermoresponsive 4D hydrogels are quite different from the above 4D thermoresponsive materials used for biomedical application, actuator, and tissue engineering. Thermosensitive parenteral hydrogels are developed using amphiphilic material, such as one-way shape-morphing material possessing both a lipophilic segment (short side -CH_2_ chain, hydrocarbon ring) and hydrophilic side chain (amine, carboxylic, -OH, and sulfate groups). This can be exemplified as poly(ethylene oxide)-poly(propylene oxide) (an amphiphilic block copolymer)-pluronic copolymer (PPO-PEO-PPO), which exhibits fluid-like consistency (low viscous liquid) at room temperature and thermal gelation (gel-like cross-link) at body temperature [32]. Polycaprolactone triol is a clinically approved biocompatible biomaterial that is frequently used to get a one-way shape-morphing 4D hierarchy scaffold using castor oil and multi-isocyanate for tissue engineering [33]. A soya bean oil epoxidized acrylate scaffold (one-way shape morphing) was generated to support the human growth bone marrow mesenchymal stem cells by fixing temporary shape at −18 °C and complete recovery at body temperature (37 °C) [30]. Methacrylated PCL (polycaprolactone) and soya bean oil epoxidized acrylate exhibited thermal responses to execute a one-way shape-morphing design of a tracheal stent and biomedical scaffold, respectively, using the stereolithography technique [30,34]. Chitosan and naïve starch (in situ pore-forming capability) were used to fabricate an orthopedic implant for one-way shape morphing (in bone-tissue engineering), and the porous scaffold was generated using two human enzymes (α-amylase and lysozyme). The reported natural in situ-forming scaffold exhibited (a) impressive mechanical strength in dry/wet states and (b) biocompatibility (L-929 fibroblast cells). The degradation study (90 days) confirmed that a porous structure was generated, which could be a potential of the methodology (salt-mediated precipitation) adopted for bone-tissue engineering [35]. The two-photon polymerization technique was implemented to fabricate enzymatically (metalloproteinase-2) degradable micro-swimmer (untethered mobile microrobot) using gelatin methacryloyl and biofunctionalized paramagnetic iron oxide (Fe_3_O_4_) nanoparticles. This method was adopted for controlled release of the loaded cargo molecule at physiological conditions using a one-way shape-morphing scaffold [36]. A magnetically responding cardiovascular implant was developed using 4D shape-morphing (one-way) materials, such as benzophenone, polylactide, and Fe_3_O_4_ [37]. Moreover, a tracheobronchial splint was designed (laser sinter) using PCL and HA (hyaluronic acid), which showed one-way response under tension (stimuli) [38]. Several authors reported chemoresponsive one-way shape-morphing products for biomedical application, such as a glucose-monitoring device (prepared by inkjet printing technique using CNT (carbon nanotube)/GOx/Pt nanoparticles) and a gastric device (prepared by ultracentrifugation and coprecipitation using poly(acryloyl 6-aminocaproic acid)/poly(methacrylic acid co-ethyl acrylate)) [39,40]. Lee at al. reported UV-responsive one-way shape morphing of a sensor prepared from poly(methacrylate)-ortho-nitrobenzyl/polydimethylsiloxane/polyethylene naphthalate [41]. 

### 5.2. Two-Way Shape Morphing 

Alginate glycerol hydrogel, tricopolymer PLA-b-PEG-b-PLA/PIPAAm-based construct (thermoresponsive roll and unroll reversible temporary shape morphing), and PEG-diacrylate exhibited two-way shape-morphing behavior in response to PH, temperature, and humidity, respectively. These were applied for skin dressing, heart failure treatment, and sensors, respectively [42,43,44]. Lv et al. fabricated a humidity-responsive hydrogel (“humidity test strip”) by the photopolymerization technique using polyethylene glycol diacrylate (PEG-DA) monomer swelled under a small humidity gradient, which resulted in spontaneous deformation and reversible movement. Moreover, authors investigated the responsible factors (exposure time and molecular weight) affecting the humidity responsiveness-based sensitivity [42]. The utility of polymer film is a universal approach, which undergoes reversible water-sorption-induced swelling, and this concept was implemented for actuation by fabricating a composite polymer film when polypyrrole and polyol-borate were combined for rapid, moisture-driven locomotion [45]. Similarly, a polymer film actuator was constructed using hydrophilic agarose and azobenzene containing photoactive PEG (polyethylene glycol), which undergoes self-actuation in response to a small humidity gradient and light [46]. 

### 5.3. Multiway Shape Morphing 

In the last decades, profound scientific progress has been inculcated to design and develop polymer-based hydrogel for directed deformation and changes in response to specific stimuli under ambient environmental conditions. However, multiplexing the system for biomedical functionality is an appealing concept to design a construct with multiple and distinct properties executed under individual stimulus. An approach to develop a multiplex build was executed by the integration of multiple and small-scale structural/compositional components on macroscopic material. This approach successfully achieved a multiple 3D-shape transformation of a planar hydrogel sheet responsive to three distinct external triggers. Authors integrated multiple structural components (small scale) with varied composition in the planar sheet gel, and each component was perfectly programmed to respond only to specific stimuli. Therien-Aubin et al. fabricated a sensor responding to multiple stimuli (CO_2_, ionic strength, pH, and temperature) by a photolithography technique, wherein poly(*N*-isopropylamide), PEG-diacrylate, poly(*N*-isopropylacrylamide-co-dimethylamino-ethyl methacrylate), and poly(hydroxyethyl acrylamide)-co-poly(*N*-isopropylacrylamide) were used as the multiway shape-morphing materials for generating swelling and contraction due to localized internal stress [47]. In the treatment of irritable bowel syndrome (IBS) and gastrointestinal cancer, patients need to take up to 16 pills daily and require suppositories and invasive treatment, resulting in more side effects (cancer chemotherapy) and rectal enema, which further reduced patient quality of life. These concerns can be taken into account by designing a suitable 4D fabrication using shape-morphing dynamic material. Controlled release of a pharmaceutical drug offers several benefits and overcomes the above-mentioned challenges. The approach of fabricating a theragripper used the integration of alternative rigid panels of biodegradable photoresponsive poly(propylene fumarate) (PPF) and deformable biocompatible PNIPAm for biphasic drug release from the polymeric layers and pores in the GI (gastrointestinal) tract in response to body temperature. Authors hypothesized that by integrating (a) the thermosensitive property of PNIPAm, (b) the high rigid strength (stiffness) of degradable PPF, (c) the controlled drug-release behavior from porous polymer, and (d) the tissue-latching ability of a photolithographically designed multifingered device, controlled and extended drug delivery can be achieved for improved therapeutic efficacy to control GIT (gastrointestinal tract) cancer and IBS [48]. Thus, Malachowski et al. fabricated a dye, mesalamine, and doxorubicin-loaded theragripper that prolonged the drug release over seven days with first-order kinetics. In vitro findings suggested improved delivery of doxorubicin as compared to a control patch. Using an in vivo model, a dye (fluorescent TG1)-loaded theragripper was endoscopically delivered to the esophagus and stomach of a pig using a catheter. Result showed a biphasic, consistent release of the dye over one week [48]. Thus, this approach may overcome the unpleasant conventional delivery methods (rectal suppositories and enemas) to treat IBS, acidic and enzyme-based drug degradation, low drug absorption, and varied transit time, thereby improving patient quality of life, reducing side effects, and avoiding unnecessary high drug introduction into the patient body (16 pills daily using conventional dosage form).

## 6. Shape-Memory Effect of Hydrogels

To differentiate swelling-induced movement from SME, a key difference between swelling-induced movement and SME is the ability of shape-memory hydrogel to fix a temporary shape that can be generated by elongation, compression, and folding on demand. Once the shape-memory hydrogel is actuated, the reversal of the applied programing transformation controls the direction of movement. It is easy to obtain a direct movement in hydrogel by anisotropic swelling initiated using a gradient polymer structure or multimaterial approaches. In multimaterial approaches, a combination of hydrogel layers with distinct swelling ability is used, such as poly(*N*-isopropylacrylamide) (PNIPAm). PNIPAm exhibited swelling-induced sol-gel/gel-sol reversible transitions [49]. Similarly, various microscopic and macroscopic shape transformations were executed using various compositions of PNIPAm, shape morphing obtained by altering pH (low to high), and loading gold nanoparticles in a hydrogel matrix for thermally-induced transformation [50,51,52]. A summary of brief findings of this shape transformation are summarized in Table 2. 

SME of synthetic hydrogels were exhibited in several polymers. The thermoresponsive SME was first investigated in a poly(acrylic acid)-based cross-linked network with a main hydrophilic chain (for water-induced swelling) and short dangling stearyl side chains. The short stearyl side chain is responsible for adopting a crystalline aggregate below transition temperature (*T_tran_*) and amorphous transformation to recover a permanent shape followed by swelling above *T_tran_* [20]. Kahn et al. combined stimuli-responsive nucleic acid bridges with thermosensitive PNIPAM chains and the systems undergone reversible solution ↔ hydrogel ↔ solid transitions [53]. Short aliphatic crystallizable side chains (16-acryloylhexadecanoic acid and 12-acryloyldodecanoic acid) can be realized for swelling-induced shape transformation in SMHs (shape-memory hydrogels) [54,55]. In contrast to these smart materials, hydrogels with oligomeric side chains (oligo(ω-pentadecalactone) (OPDL) and oligo(tetrahydrofuran) (OTHF)) are capable of exhibiting permanent shape transformation by swell switching but independent of temperature even above *T_tran_* [56]. Similarly, chemically cross-linked PEG hydrogels and interpenetrating side chains of PVA (polyvinyl alcohol) hydrogels can be fabricated into crystalline shape-morphing domains by freeze/thaw cycles to get stabilized temporary shapes [57]. Hydrogel allows diffusion of small molecules that may serve as the trigger for the shape-memory effect. The molecular switches (hydrogen bonding, dipole–dipole interaction, and ion complexation) can fix a temporary shape of hydrogels. However, these switches can be cleaved by pH changes, redox reaction, and complexing agent. A hydrogel containing carboxylic acid (functional group in monomer) was enabled to fix its temporary shape by the addition of Ca^+2^ solution, followed by reversible shape recovery by cleaving Ca^+2^ –carboxylic complexation using a complexing agent [58]. Notably, a response of a hydrogel may be achieved using a single type of temporary cross-link (PVA and boronic acid to give a boronate ester bond as a reversible cross-link), sensitive to only one type of stimulus or using two different types of temporary cross-links (copolymerization of acrylamide and acrylic acid with low cationic alkyl short chain), sensitive to individual stimuli [59,60]. Triple-shape hydrogels (TSHs) permit two types of steps during shape switching in response to stimuli (swelling-mediated induction). This type of hydrogel can be realized by adding two different types of side chains (crystallizable) in a hydrophilic cross-link network, such as a copolymer network consisting of oligo (ethylene glycol) (OEG)-cross-linked *N*-vinylpyrrolidone (NVP) as a backbone chain and oPDL (oligo(ω-pentadecalactone)) and OCL (oligo(ε-caprolactone)) or OTHF (oligotetrahydrofuran) as side chains [61]. Various examples of other types of cleaving agents and SMHs are described in Table 2 [62,63].

The natural biopolymer possessing natural self-organization is often responsive to multiple stimuli for designing reversible, temporary cross-links. However, the self-organization ability is critical to control, yet challenging, which can be resolved by constructing a suitable polymer architecture network followed by manipulating the cross-link density. The cross-link density must be in optimal range as high density results in the hindrance of self-organization behavior of SMHs. The self-organization behavior of natural biopolymer is based on two molecular switches, including (a) H-bonding interaction and (b) noncovalent interaction. A triplex helix of a polypeptide was obtained as a reversible temporary cross-link using H-bonding interaction (molecular switch) followed by cooling, and subsequently, permanent recovery was achieved by H-bond cleaving (dissolving triple helices) on melting (heating) [64]. Similarly, triple helices of gelatin protein were executed for temporary reversible cross-link using graphene oxide (as molecular switch) and subsequently recovered by near-IR irradiation (graphene oxide as IR-absorbing molecule) [65]. Despite the concept of natural self-organization in the natural biopolymer, conventional switches can be implemented for reversible temporary cross-link, such as thermosensitive hydrophobic interaction and pH-sensitive interaction. Ionic liquid was incorporated in polysaccharide-based xanthum gum to induce reversible temporary switch shape (cross-links) in response to thermosensitive intermolecular interaction [66]. Similarly, incorporated PBA (dynamic phenylboronic acid)–catechol bonds in alginate hydrogel result in a permanent netpoint through pH-sensitive ionic interaction between Ca^+2^ ion and alginate [67]. Notably, a dynamic phenylboronic acid (PBA)–catechol bond was established as a temporary cross-link at alkaline pH, whereas this bond was cleaved (dissociation of the bond) at acidic pH for permanent recovery. These tailored netpoint-based, biopolymeric hydrogel respond to multistimuli as the “PBA–diol ester bonds” could be reversibly cleaved by sugar [68]. A double network obtained from a cross-linked PAAm and PBA-grafted PVA-alginate, allowed formation and reversible dissociation of two independent temporary netpoints (noninterfering temporary cross-links) [69]. Thus, these described concepts resulted in designing biopolymer-based SMHs. 

The shape-morphing hydrogel exhibits dimensional changes (swelling and de-swelling), depending upon types of stimuli, and these changes result in (a) interference with the directed movement, (b) limited recovery, and (c) macroscopic effect. Implementing the concept of superstructure in hydrogel results in improved functionality in these various materials-based techniques as tabulated in Table 2 and Table 3. A superstructure facilitated the diffusion of ions, molecules, and nutrients at a microscopic level within the system under provided stimuli (water, mechanical compression, and temperature). Several techniques (salt leaching, gas foaming, cross-linking agents, and shape-switching agents) to make porous and nonporous polymer hydrogel have been reported for improved functionality of these polymeric hydrogel (Table 2) [70,71,72,73,74]. Overall, this report suggested that the applied external switching agents may generate reversible temporary cross-links with directed movement though implementation of structural complexity. These improved functionalities are required for increased mechanical strength in nonporous hydrogel, increased recovery performance, and directed movement. For example, TiO_2_ serving as a coordinate cross-linking agent improved 1.55 and 3.1 times the tensile strength and the compression strength of nanocomposite hydrogel, respectively, when it was allowed for a strong interaction between TiO_2_ and poly[(acrylic acid)-co-(*N,N*-dimethylacrylamide)] [74]. 

## 7. Classification of SMH-Based on Stimulus: Stimuli Responsive SMHs

### 7.1. Aqueous (Water)-Sensitive SMHs

It is worthwhile to mention that the details of response-shape-morphing transformation mechanisms were described in previous sections describing several examples. In this section, water as a potential switch-shape inducer for temporary cross-links to fabricate SMHs is discussed. It is a well-established fact that hydrogel is capable of absorbing a substantial amount of water and acts as an aqueous buffer and physiological fluid for swelling (significant volume change). It exhibits slow diffusion of small molecules, ions, nutrients, and oxygen in the environment. Moreover, hydrogel elicits great de-swelling upon dehydration (loss of water), resulting in volume shrink. Thus, hydrogel possessing swelling and de-swelling behaviors has been implemented to fabricate 4D shape-memory hydrogels (SMHs) in response to directed stimuli for desired functionalities. Several authors reported different concepts of swelling behavior to fabricate 4D SMHs, such as (a) anisotropic swelling, (b) polymers with different degrees of swelling, and (c) superstructure hydrogels using gas bubbles in a gel matrix while processing [86,87,88,89]. A summary of various findings is presented in Table 4, wherein various techniques and polymer materials have been exploited to fabricate water-sensitive hydrogels for desired applications [86,87,88,89,90,91,92,93,94,95,96,97,98,99,100,101,102,103]. 

### 7.2. Thermosensitive SMHs

Temperature is the most widely played stimuli for developing thermoresponsive shape-memory fabrication in hydrogels. A number of authors reported thermosensitive hydrogels for vital applications in biomedical, tissue engineering, clinical surgery, and engineering technology in the literature (Figure 3 and Figure 4). It is a well-established fact that thermosensitive hydrogel undergoes reversible temporary volume change due to the expansion or collapse of the polymer chain in the aqueous solvent/physiological fluid/water at the critical temperature (coil-globule transition) [104,105]. Thus, hydrogels exhibit different shape-transformation properties when exposed to a temperature above or below the lower critical solution temperature (LCST). Several findings were compiled in this context and shortly described in Table 4 [90,91,92,93]. There are few examples of biopolymer exhibiting thermoresponsive shape transformation above or below the upper critical solution temperature (UCST) of the hydrogels. These are agarose and gelatin as biopolymers, which execute different phase behaviors, such as homogeneous solution or phase separation above and below the UCST, respectively. This results in dramatic variations in optical and mechanical properties of the hydrogels after exposure to temperature below or above UCST of the hydrogels [94,106,107]. 

### 7.3. Chemically Sensitive SMHs

Several hydrogels are responsive to chemical stimuli (ions, biomolecules, phosphate buffer solution, ethylenediamine tetraacetic acid, CaCl_2_ solution, pH, acidic solution, alkaline solution, water, and organic solvents, etc.) for shape switching with targeted functionalities. Agarose, alginate, gelatin, hyaluronic acid, PAAm (polyacrylamide), poly(acrylic acid), and (PAAc) have been exploited to fabricate chemoresponsive hydrogels for various biomedical applications as compiled in Table 4. The mechanism for their responsive transformation to chemical stimuli is related with association (triggered cross-linking) and dissociation (bond cleaving) of the hydrophilic/lipophilic side chain of the polymers or ion-induced modification/interaction in main chain as described before [108,109,110,111]. These polymer chains exhibit cross-linking-based shape morphing due to electrostatic interaction with chemical stimuli such ions, solvent, and molecules. Type and concentration of ions interfere with the strength of electrostatic interaction and the properties of hydrogels. Hydrogels containing a large number of carboxylic functional groups in the short dangling chain are highly responsive to pH change in the surroundings (inside or outside body), resulting in shape morphing for various targeted functionalities (clinical and nonclinical applications). This pH-triggered shape transformation can be applied for drug delivery. Thus, reducing the ionization pH threshold up to the physiological pH in a modified hydrogel system would result in a modified drug release to the targeted site for improved therapeutic efficacy and reduced plasma drug fluctuation [95,96]. Some miscellaneous stimuli, such as magnetic field, near-IR (infrared) irradiation, and electrical, were used to design 4D hydrogels for biomedical application. Accelerated IR-radiation-based heating that bends the alginate-based PDA scaffold is an excellent strategy, which can be implemented to fabricate a self-folding 4D cell-laden construct/design for functional and dynamic artificial tissue or organ as a lifesaving emergency fabricate (an alternative to tissue or organ transplantation) [98,99,100]. Moreover, multistimuli-based responsive hydrogels are another promising and potential approach with high functionality and biomedical application in tissue engineering [101,102]. 

### 7.4. Comparative Analysis of 3D- and 4D-Printing Techniques

Three-dimensional technique is a precursor to 4D-printing/bioprinting technique. Both techniques are associated with certain advantages and disadvantages as shown in Table 5. In general, they differ in terms of printing materials, printers, product quality, responsiveness to certain stimuli (physical, chemical, and biological), printing process speed, expenses for printing or constructed object/scaffold/construct, and shape-morphing behavior. All of these are included in Table 5 to investigate a comparative study between these two techniques [112,113]. However, there are certain differences that are not related to printability. These are expenses, regular program-based upgrades in used software, intellectual property rights, and environmental issues. The EPA (Environmental Protection Agency) has control over the safety concerns of the materials and organic solvents that have adverse impact on the ecosystem (soil, aquatic system, and air pollution). Uncontrolled drainage of toxic materials exposed to aquatic or soil systems may result in serious adverse effects on flora and fauna. 

### 7.5. Overcoming Rheological Limitations of 3D-/4D-Printing Hydrogels

Hydrogel standalone is not applicable for 3D/4D bioprinting in tissue engineering, despite having several benefits, such as good swelling and biocompatibility. This is due to the rigid and highly viscous nature of hydrogel, which is responsible for poor extrusion through the printing nozzle and poor flow. This rheological issue limits biomedical application of several biocompatible hydrogel-forming materials, such as carbopol, gelatin, alginate, collagen, cellulose and cellulose derivative, and hyaluronic acids. However, several recent advancements have claimed improved rheological behavior of these polymer-based hydrogels for biomedical and tissue engineering. In general, these approaches were functionalization, derived form of polymer, nanocarrier, or a combination of polymers to achieve desired flow of 3D-/4D-printing/bioprinting ink. 

Bioinks are formulations containing cells, sometimes materials, to be processed under automated biofabrication technology for printing cells directly as spheroids or organelles [114]. It is noteworthy that shear and extensional stress have significant impact on the cells’ viability within a hydrogel matrix. Thus, introduction of cells in the matrix can cause substantial changes in the ink material that they are printed in with implications on cell density and final shape fidelity. The reports of complete bioink (cells and materials) rheology are infrequent in literature due to the time and cost of expanding cell culture. Considering cells as a spherical particle (with a certain value of volume) has very low impact on rheological behavior of bioink due to poor particle–particle interaction (volume fraction at ˂40% is Newtonian flow) [115]. Several authors reported manipulation of temperature, viscosity, and composition of materials followed by functionalized materials that resulted in desired rheological properties of hydrogel for hydrogel-based bioprinting. In this section, we addressed several approaches taken into account to overcome rheological issues of hydrogel-based printing technique for constructing 3D/4D objects. 

***Nature of gelling material/polymer:*** In polymer solutions, there are three types of interactions: (a) solvent–solvent interaction, (b) polymer–polymer interaction, and (c) polymer–solvent interaction. In the polymer–solvent interaction, there is an interaction energy required to solubilize polymer in the solvent (Flory-Huggins parameter, x). These polymer–solvent interactions are critical for the swelling behavior of hydrogel as the volume fraction and materials chemistry dramatically affect water uptake and rheological properties [116]. The gelling behavior of hydrogel can be controlled by using nonionic and ionic polymers for hydrogel, as the thermodynamic, nonionic, polymer–solvent interaction and contractive force of hydrogel are balanced to achieve an equilibrium swelling state with minimum viscosity and high flowability. Moreover, postprinting swelling may cause reduced nutrients and diffusion of oxygen into hydrogel, which can be overcome by strong cross-linking hydrogels of charged polymers, such as negatively charged HAMA (methacrylated hyaluronic acid) and positively charged chitosan (charge compensation-induced water expansion) [117].

***Cross-linking of polymer***: Hydrogel-forming polymer may be induced for cross-linking under physical (noncovalent bonding), chemical, or a combination of these two methods. However, the photo-induced cross-linking method is the most commonly used in hydrogel for cell viability and shape fidelity in 3D/4D bioprinting. Thus, the most popular examples are methacrylated gelatin (GelMA) and PEGDA (polyethylene glycol-diacrylate) used as bioink for simplicity in manufacturing, extrusion, and light-driven cross-linking [116]. 

***Dynamic bioink***: Dynamic chemistry introduced shear thinning under stress and self-healing after removal of applied stress due to reversible bonds (ionic and covalent bonds) in materials used in hydrogel. The concept of supermolecular chemistry described the association of molecules through noncovalent interactions (hydrogen bonding, pie–pie interaction, transition metal complex, and ionic and hydrophobic interactions) for designing bioink formulations of hydrogel responsive to specific stimuli. These materials are supramolecular polymers, guest–host complex, supramolecular polymer network, and self-assembled architecture [118,119,120]. Dynamic covalent cross-linking was another method to obtain tuned bioink (aldehyde-based silica nanoparticles and oxidized alginates) for high cell and suitable rheological properties [121]. 

***Particulate and nanocomposite bioink***: Hydrogel structured (spherical and stranded microgel) at the microscale and nanoscale are used as the bioink for strong shear thinning and low thixotropic behavior. Nano-to-micron-sized particles have also been used to improve printability of 3D/4D bioink. Aqueous solution of laponite (nanoclay with plate-like morphology possessing a negative charge at the surface and positive charges at the edges) readily forms structured fluid, which adds beneficial effects to rheological properties of bioink [122]. 

***Polymer blends and additive***: The rheological behavior of bioink can be improved by blending one polymer at low concentration with another polymer. This was achieved in methacrylated hyaluronic acid-based hydrogel (HAMA), commonly used in tissue engineering. HAMA alone (2.5% *w*/*v*) was observed to have low viscosity and poor shape fidelity postprinting. However, the shape fidelity was improved by blending with 5% *w*/*v* gelatin for rapid printing in a low temperature bed (15 °C). The HAMA gel was photocross-linked at 37 °C (during culture growth). Thus, gelatin was added to a variety of methacrylated polymers (alginate, gelatin, chondroitin sulfate, chitosan, dextran, and heparin) for improved cell viability and rheological behavior of hydrogel [123]. 

**Figure 5:**
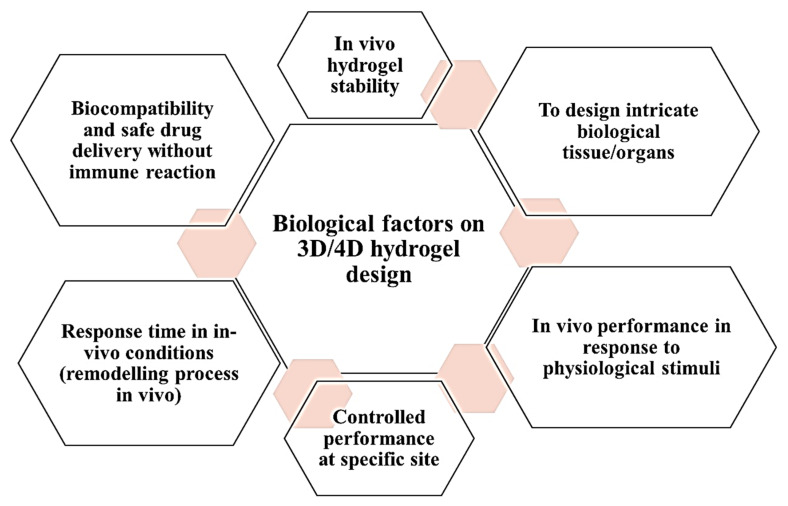
Biological factors responsible for controlling in vivo performance and other factors for 3D-/4D-bioprinted hydrogel-based scaffold/construct/design.

Notably, 3D-/4D-printed/bioprinted scaffolds are considered to mimic in vivo performance for the designed tissue or organs. However, there are several factors being affected due to in vivo physiological conditions or in vivo environment. These are physiological pH, extracellular matrix composition and properties, intra- and extracellular enzymes, and immune responses. The designed construct should have biocompatibility and be free of immune reaction. Thus, these biological factors are responsible for changing the in vivo stability, hypersensitivity reaction, varied response time, reversibility, drug release pattern, and remodeling process (in vivo) of 3D/4D scaffolds/objects/constructs as shown in Figure 5. 

## 8. Challenges and Future Perspective

In the last decades, progressive and proficient advancement of 3D material-based printing technology opened a new era of 4D-printing technology with wide biomedical, tissue engineering, and clinical applications. The generated 4D sophisticated, dynamic structure achieved prescribed functionality, accuracy, substantial resolution, and versatility. The most critical challenge for 3D printing is to construct hollow tubular structures (blood vessels, capillaries, and venules) with high resolution and versatility. The 4D technological products have emerged with positive impacts on various domains of research, such as drug delivery, diagnosis, actuation, tissue engineering, nerve soft grafts, biosensors, theragrippers, biocompatible implants, nanochips, and stents. Despite having various advantages, 4D technology is still associated with several challenges and needs to be addressed in this review. Four-dimensional-printing materials and technology are still at the exploration stage, as evidenced by the unavailability of specifically 4D printers in the market. Moreover, the current technology must be substantially improved with high precision for developing medical devices. The present 4D technology and precision do not meet these criteria.

**Figure 6:**
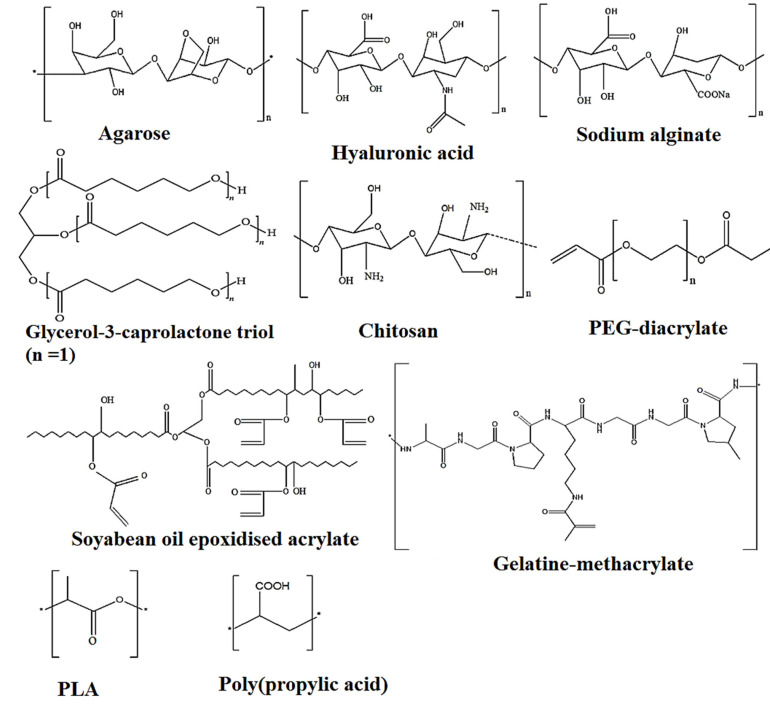
Chemical structures of a few biocompatible biopolymers executing 4D shape-morphing behavior in response to stimuli.

The biological environment varies patient to patient in terms of complexity, dynamics, and responsiveness. The developed 4D-printed construct should be adapted in the microenvironment of the patient body; for instance, a microfluidic system generating an optimal plate to reach cell biological potential to produce a functional tissue [124]. Considering dynamic, shape-morphing materials, advanced polymers, biopolymers, and nanocomposites, these are capable of changing their shape and functionality in response to subjected stimuli. These materials are either synthetic, semisynthetic (functionalized), or natural. They have to be nontoxic, biocompatible, long-lasting, and nonimmunogenic and have optimal mechanical strength. Thus, only limited materials possess these aforementioned criteria for clinical application. Moreover, most of the available smart materials respond to only a single stimulus, such as temperature, and thus restrict further clinical and biomedical application. In case of 3D-bioprinting materials, limited natural, synthetic, and functionalized biopolymers/polymers are available. For instance, these are enlisted as protein (gelatin), polysaccharides (starch, chitosan, agarose, alginate, hyaluronic acid), acrylate, poly(lactide), poly(caprolactone), castor oil, functionalized (soya bean oil epoxidized acrylate), PEG, and graphene (Figure 6).

Despite advancement in 4D materials for developing soft tissue with high complexity, no commercial 4D printer is available so far due to lack of clinical trial data, and the maturation of the printed tissue is incomplete. Therefore, a postprinting process is required to justify the maturation of the printed tissue by the cellular coating and cell organization [125].

Furthermore, the functional processes of the hydrogel-forming materials exhibit limitations, such as lack of fast response and recovery to stimuli. Parenteral biocompatible scaffolds prepared using PCL and soya bean oil epoxidized acrylate polymers exhibit complete recovery at or below physiological body temperature (Figure 6). However, the morphological changes over time and functionality of the cells must be monitored, which still remain to be investigated. Most sophisticated in vivo, ex vivo, and in vitro analysis data are still required to address the possible challenges/problems for improved therapeutic application, tissue engineering, and clinical application of these biomaterials. Currently, 4D scaffolds only exert a single type of deformation on the cells and the prolonged effect is minimal due to mechanical stimuli. So, studies are still needed to identify various suitable shape-memory morphing polymers and nanocomposites for programmed shape recovery.

There are several new advancements in 3D- and 4D-printing constructs, such as implementing adaptive 3D-/4D-printing techniques, metamaterials used in 4D-based objects, and applying topology optimization (TO) tool-based 3D/4D fabrications. These advancements might achieve better patient welfare outcomes. The concept of topology optimization (finite elemental analysis) and 3D printing further improved patient clinical outcomes (increased patient comfort and fast recovery) [126,127]. Moreover, topology optimization (TO) along with 4D printing became a powerful digital tool to fabricate optimal internal architectures for the efficient performance of the soft actuator to deliver drugs in the delicate microenvironment of body tissue or engineered regenerated tissue [127,128]. Thus, Zolfagharian et al. developed a TO-optimized, 4D-printed soft actuator (using a 3D bioprinter and polyelectrolyte hydrogel) having the full potential of actuation (due to porous material) with multiple functionality (maximal free-bending deformation) [128]. In 2020, Zolfagharian and colleagues reviewed a comprehensive report on adaptive 4D-printing systems wherein there were informative recent progressive developments on control-based 4D printing with highly versatile, multidisciplinary applications. Such systems were responsive to environmental dynamic situations and uncertainties as nature does, and adaptive metamaterials opened a new domain for multifunctionality of 4D printing/bioprinting [129,130].

Recently, commonly used energy sources, such as lithium batteries and supercapacitors, have been recognized as emerging prime power sources. Therefore, Zhu et al. reviewed 3D-printed functional nanomaterials for energy storage with comprehensive findings [131]. Further, development in ink materials opened a new trending research for 3D and 4D printing: the development of colloidal nanoparticle ink for 3D printing functional devices. Zeng and Zhang compiled a comprehensive review of this colloidal nanoparticle ink for 1D-, 2D-, 3D-, and 4D-printing functional devices [132,133]. Thus, shear thinning as a required nature of ink limits smooth extrusion of bioink, such as biological hydrogels. These nanoparticle-based inks may be optional for biological hydrogels in 3D- and 4D-printing purposes. Notably, Choi et al. combined self-healing hydrogel and self-healing ferrogel to fabricate a 3D-printed, dynamic tissue scaffold that functioned as a biological extracellular matrix (ECM) [134]. The constructed scaffold mimicked (functionally) the biological ECM under physiological conditions. Graphene and graphene quantum dot-based multifunctional sensors and colloidal nanosurfactants for 3D printing are still challenging for implementation in 4D and 3D bioprinting for tissue engineering [135,136]. 

## Figures and Tables

**Table 1:** A comparative analysis of conventional 3D hydrogel printing, 4D hydrogel printing, and 4D, SMP-based printing [7,8,9,10,11,12,13].

S. No	Features	3D Hydrogel Printing	4D Hydrogel Printing	4D, SMP-Based Printing
1	Fabrication process	Built in layer by layer on 2D constructs or designs from bottom to top (step-up process)	Transform 3D designs or constructs under certain external stimuli using smart materials	Transform 3D designs or constructs under certain external stimuli using shape-memory polymers
2	Materials	Biomaterial, hydrophilic material, proteins, nanomaterials, plastics	Physiological responsive biomolecules, thermoresponsive hydrophilic material, chemoresponsive proteins, stimuli responsive shape-morphing material	Physiological responsive polymers, thermoresponsive hydrophilic and lipophilic material, chemoresponsive polymers, stimuli responsive shape-morphing polymers
3	Deformation characterization	Rigid, stiff, and no flexibility	Flexible, high-swelling capability	
4	Compatibility	Incompatible	Biocompatible	Incompatible
5	Toughness	No tunable toughness	Tunable toughness	Moderate toughness
6	Water content	Low	High	Low
7	Cost	High	Low	High
8	Shape	No change over time in response to trigger stimuli in the environment	Change occurs over time in response to trigger stimuli (physical, chemical, and biological stimuli) in the surrounding environment	Change occurs over time in response to trigger stimuli in the surrounding environment
9	Programmable material	No use of advanced smart and programmable materials	Use of smart, shape-morphing, and programmable materials	Use of smart, shape-morphing polymers, and programmable polymer materials
10	Limitations	Most of these materials resulted in printed objects that were inanimate or dead, restricting their applications when time-evolving, shape transformation is needed. Low-resolution printing observed Low switching and recovery response	In practice, the development of fabrication at the microlevel with fast speed response is still critical for targeted drug delivery and bioengineering. Four dimen- sional printing of hydrogel with high complexity and multiple freedoms of shape morphing remains to be explored. Time scale is critical to control below minimum scale. High switching and recovery response	Due to low sustainability in wet environments, high rigidity, low material permeability, and probable chances of biological incompatibility due to polymer degradation over time, it cannot completely replace soft hydrophilic materials. High switching and recovery response
11	Advantages	Faster than 2D and 1D printing	Apparent transformation of 3D constructs whenever time-evolving shape transformation is required Faster printing than 3D, 2D, and 1D High-resolution printing possible using 4D hydrogels	Apparent transformation of 3D constructs whenever time-evolving shape transformation is required Faster printing than 3D, 2D, and 1D High-resolution printing possible using 4D hydrogels

**Table 2:** Description of various types of shape-memory effects (SMEs), molecular switching for temporary shape fixations, and cleaving agents in hydrogels.

Swelling-Induced Movement	SME of Synthetic Hydrogels	SME in Biopolymer Hydrogels	SME in Hydrogel Microstructures
Direct movement can be achieved by anisotropic swelling	Molecular switches for temporary shape fixing are not integrated with the main chain of polymer cross-link network.	The biopolymer possesses natural self-organization for reversible temporary cross-links in response to various natural stimuli.	Improved functionality is achieved by increasing structural complexity in porous, nonporous, and hybrid hydrogels.
Example: gradient polymer network structure or multimaterial approaches	These shape transformations (reversible) can be achieved through crystallizable side chains (oligomeric), short chains, complexing functional groups, and host–guest interactions.	The self-organization due to noncovalent interaction (H-bonding and ionic interaction) is critical to control (challenging). So, a suitable polymer cross-link is required to form biopolymer-based SMHs.	Hydrogel exhibits swelling or de-swelling depending on types of stimuli, and the dimensional change may interfere with the directed movement, recovery, and functionality.
Polymer combination: combination of hydrogel layers with distinct swelling	Hydrogel displays a condition that permits fast diffusion of small molecules, and these may serve as a trigger for SME.	A cross-link density plays a vital role in influencing flexibility, elasticity, mechanical strength, and swellability in newly designed biopolymer-based SMHs.	Leaching technique, gas foaming, and cross-linking reactions in emulsion results in superstructure formation largely by integration of interconnecting pores, which subsequently leads to minimized volume change after swelling or de-swelling.
Poly (*N*-isopropylacrylamide) as PNIPAm exhibited sol-gel/gel-sol swelling-induced reversible transition	Molecular switches are hydrogen bonding, ion complexation, and dipole–dipole interaction as an alternative to crystallizable domains for temporary shape fixing.	A cross-link density should not be too high for developing biopolymer-based SMHs, which has negative impact on shape-memory transition behavior of SMHs.	This superstructure facilitates movement of ions, molecules, water, nutrients, and oxygen from the inside to the outside of a cross-link network in response to heat, water, or a combination of both, resulting in rapid response to the applied stimuli.
Different PNIPAm composition shows planar to helical transformation upon changes of ionic strength or pH	Molecular switches can be cleaved by (a) pH change, (b) complexing agent, (c) redox reaction, (d) acidification, (e) photoacid (UV) generation (PAG), (f) CO_2_, and (g) cerium ammonium nitrate (CAN).	High density, amount, and strength of the noncovalent interaction result in hindrance in self-organization behavior of the natural bi opolymer to various stimuli for shape transformation.	A crystallizable switching domain (short side chain) of OCL integrates with the hydrophilic network structure of NVP (*N*-vinyl pyrrolidone) and OEG (oligo(ethylene glycol)) to generate a porous microstructure by a salt-leaching technique that is recovered by heating [70].
PNIPAm-based hydrogel loaded with gold NPs execute shape effect upon thermal induction	Redox reaction in hydrogel is used for selective interaction between cyclodextrin and ferrocene (host–guest), whereas cerium ammonium nitrate (CAN) is used for recovery from oxidation-based temporary cross-link.	Commonly, two natural biopolymers were used: (a) polysaccharides and (b) polypeptides for biopolymer-based SMHs using H-bonding and ionic interaction as molecular switches.	A 3D porous network cross-link of gelatin hydrogen is obtained by decreasing *T_g_* (glass transition temperature) of the system after water addition that exhibits remarkable shape-switching functionality [71].
Cysteine-rich amino acid sequences generate *i*-motif structure in low pH-induced self-assembly and subsequent transition to “quasi-liquid state” at high pH (~8.0) [49,50,51,52]	The physical cross-link boronate ester could be cleaved by lowering/raising pH and indirect heating (ultrasound). Another TSH is based on poly[acrylonitrile-co-(2-methacryloyloxyethyl phosphorylcholine], where the nitrile (CN) ions create dipole–dipole interactions (CN-Zn-CN and CN-CN) in the presence of low zinc ions. It can be reversed in high zinc concentration. TSHs can be implemented for multistimuli responsive hydrogel by incorporating photo- and pH-sensitive moieties in a copolymer cross-link network.	A thermally induced polypeptide-based hydrogel is formed by H-bonding for triplex helix shape on cooling, then recovered by cleaving H-bonding using heat (melting). Similarly, a triple helix temporary shape was designed using gelatin containing graphene oxide and subsequently recovered permanently by near-IR irradiation and dissipation of thermal energy. Conventional molecular switches: pH changes, thermoresponsive stimuli, and hydrophobic interactions, can also be implemented for biopolymer-based SMHs. Example 1: xanthum gum with ionic liquid	NIPAm, heterbifunctional cross-linker and 4-hydroxybutyl acrylate provide a well-defined gradient pore structure, and these can be implemented to generate structural complexity [72]. A gradient porous morphology is achieved by precipitating the main chain components using a suitable cross-linker due to gravitational distribution of netpoints. This gradient pore architecture permits the directed movement of hydrogel by anisotropic swelling, and temporary shape is recovered by heating. Original shape is recovered by the addition of water in the system. Ultrasound can also be used as an indirect heat source for implementing structural complexity [73].
	Acrylic acid-derived copolymer cross-link networks contain CD as host group and azophenyl derivative as guest group that form a temporary cross-link due to host–guest interaction, which are sensitive to light and pH [62,63].	Example 2: For a permanent netpoint, a reversible PBA–catechol bond is incorporated in alginate-based hydrogel in response to pH stimuli through ionic interaction between alginate and Ca^+2^ ion. [64,69]	Nonporous polymer hydrogel possesses weak mechanical strength, low elongation, and high brittleness, which can be improved using coordinating cross-linking agent (TiO_2_) for hybrid hydrogel. Example: poly[(acrylic acid)-co-(*N,N*-dimethylacrylamide)]-based hybrid hydrogel [74].

**Table 3:** Various techniques/processes, pros and cons, and materials used to fabricate 4D constructs.

Terms		Processes					
	Material Extrusion	Vat Polymerization	Powder Bed Fusion	Material Jetting	Binder Jetting	Sheet Lamination	Directed Energy Deposition
**Technique**	DIW and FDM	SLA, 2PP, and DLP	DMLS and polymer SLS	PolyJet-printing	BJ	LOM and UAM	EBM
**Raw material**	PNIPAm, PAAm, agarose, cellulose, alginate, HA, PLA, ABS, PC, PA	Typical polymers: acrylate/epoxide	Bending of unique polymers (PA-12/PEEK)	Acrylate polymers	PLA, starch, ceramics, silicon carbide	PVC polymer and paper, LIG (laser-induced graphene oxide)	Titanium, cobalt–chrome alloys, β-type Ti2448 alloy
**Form**	Solid filament and liquid ink (shear thinning behavior)	Liquid photopolymer	Blend powder	Melted liquids	Crystalline solid SiC and liquids	Solid sheets, graphene foam	Solid wire or powder
**Advantages**	Economic, versatile and facile Customizable Wide range of materials Controlled deposition of molten feed material Printing up to 400 °C	Rapid printing High resolution (50–100 µm) Wide range of materials Low imaging energy Excellent surface High precision (0.1–5 µm) Multiphoton lithography	Stable No postcuring step No support required Poor recycling High complexity High resolution (50–100 µm) Less anisotropic High mechanical strength	Low waste Wide range of materials Fast printing	High speed Support structure included High resolution (100 µm) Multimaterial additive manufacturing High thermal properties	High speed without support Low warping and internal stress High anisotropy Good electrical conductivity and mechanical strength	High efficiency for repair Add-on features Suitable for large components High mechanical strength Low Young’s modulus
**Disadvantages**	Poor resolution (100–150 µm). Anisotropic print High computation cost Low volume needed	Poor mechanical strength Postcuring required Supporting structure needed	Expensive Rough finished surface	Unable to recycle Postprocessing (causing damage) Low resolution Low viscosity ink	Rough surface Low viscous ink required Low temperature required	Limited materials Noxious fumes Low resolution (100–150 µm)	Poor dimensional accuracy Limited choice for materials Rough strut surface
**Controlled** **parameter**	Ink composition, rheology, and printing variables	-	-	-	-	-	Material property, stress
**Largest build** **volume**	200 × 200 × 200 mm^3^ 1005 × 1005 × 1005 mm^3^	250 × 250 × 250 mm^3^ 800 × 330 × 400 mm^3^	250 × 250 × 250 mm^3^ 1400 × 1400 × 500 mm^3^	300 × 200 × 150 mm^3^ 1000 × 800 × 500 mm^3^	200 × 250 × 200 mm^3^ 1000 × 600 × 500 mm^3^	300 × 200 × 150 mm^3^ 170 × 220 × 145 mm^3^	Strut thickness (460–632 µm)
**Ref**	[75,76,77]	[78,79]	[78,80]	[78]	[78,81]	[78,82,83]	[78,84,85]

Note: DIW: Direct ink writing, FDM: Fusion deposition modeling, SLA: Stereolithography, DLP: Digital light processing, DMLS: Direct metal laser sintering, SLS: Selective laser sintering, LOM: Laminated object manufacturing, UAM: Ultrasonic additive manufacturing, BJ: Binder Jetting, EBM: Electron beam melting, HA: Hyaluronic acid, 2PP: Multiphoton polymerization, PVC: Polyvinyl chloride, PLA: polylactide, ABS: Acrylonitrile-butadiene-styrene copolymer, PC: Polycarbonate, PA: Polyamide, PAAM: polyacrylamide.

**Table 4:** Various stimuli responsive, shape-memory hydrogels (SMHs) [72,73,74,75,76].

Type of Hydrogels	Technique and Materials/Polymers	Major Findings	References
Water-responsive hydrogel	Copolymer between PEG and PGTM (poly(tetramethylene glycol))	Constructed hygroscopic robotics using both inactive and active (moisture-sensitive) layers of polymer. Thus, beeswax and PEG-PGTM served as active and inactive layers, respectively. A fast hygroscopic expansion of polymer occurred as a result of H-bonding formation and cleavage during the humidification and dehumidification processes. The actuator bent towards inactive layer reversibly (4D aircraft model) in response to humidity gain of 20–80%.	[86]
	Cotton-derived pulp fibers and CMC (caroboxymethyl cellulose) Extrusion technique	Similar concept was utilized to design cellulose hydrogel composite ink. At the base of composite petal, a circular layer of CMC/HEC (hydroxyl ethyl cellulose) without pulp fiber was used. The petal layer swelled and moved out of plane due to difference in swelling ratios of both layers upon dehydration. Recovery took place during hydration at room temperature.	[87]
	Soft *N,N*-dimethyl acrylamide and stiff nanofibrillated cellulose (NFC)	Authors implemented anisotropic behavior of hydrogel during swelling. The stiff nanofibrillated cellulose was aligned in the printing path owing to shear stress or strain. This resulted in longitudinal and transverse swelling movement (curve movement).	[88]
	Polyethylene glycol diacrylate (PEG400DA) and 2-hydroxy ethyl methacrylate (HEMA).	Ji et al. used the same concept of asymmetrical swelling using two polymers to fabricate hydrogel possessing anisotropic swelling behavior. The orientation of grooves printed on the one side of the printing strip governed the direction of the asymmetrical swelling direction. The perpendicular grooves spontaneously deformed toward a circle in response to water-based swelling, whereas the inclined grooves forced the strip to twist and created a helical bent. Recovery occurred in air after removing water.	[89]
	Alginate, CaCl_2_ solution, and gas bubbles Microfluidic technology	Authors developed controlled alginate-based hydrogel microfiber embedded with gas bubble exhibiting shape switching in response to dehydration. Alginate matrix was cross-linked with calcium ion and trapped air bubbles to generate pores. The bubble pattern was controlled by tuning the middle flow rate and the gas flow rate. These bubbles were evenly distributed in the matrix to create butterfly shape. The bending curvature of the fiber was linearly related with the gas flow speed due to the asymmetrical shrinkage principle. The design was recovered in hydration.	[65]
Thermosensitive hydrogels	PNIPAm	This is the most studied thermoresponsive polymer, having a LCST (lower critical solution temperature) value of ~32 °C. The polymeric networks collapsed to expel water at an aqueous temperature ˃ LCST, resulting in volume shrinkage, whereas they swelled to absorb water at an aqueous temperature ˂ LCST resulting in volume expansion.	[90,91]
	PNIPAm and alginate	The developed ionic covalent entanglement hydrogel responsive to temperature. Alginate improved mechanical property, whereas PNIPAm was able to produce shape transformation in response to temperature. A thermally controlled microvalve was fabricated by submerging (1.4 ml/s as flow rate of water within matrix) in cold water (20 °C) and subsequently heating up to 60 °C (above LCST of PNIPAm) to close the opening. This can be reversed (recovered) by decreasing the temperature up to 20 °C.	[92]
	PNIPAm and PHEMA (Polyhydroxyethylmethacrylate)	Authors fabricated a cubic construct responsive to heat/temperature. PHEMA was nonresponsive to temperature, whereas PNIPAm responded to heating stimuli for reversible transition. The PNIPAm-based top layer of hydrogel swelled more than the PHEMA (bottom transparent layer) in water (20 °C), which resulted in bending of the bilayer towards the PHEMA side and vice versa at 60 °C (due to coil-to-globule transition).	[93]
	Agarose and acrylamide In situ polymerization	Guo et al. constructed 4D-printing hydrogel by in situ polymerization of acrylamide and agarose. The agarose has the UCST value of about 35 °C, which showed homogenous and deformed cross-link design above the UCST. Similarly, the agarose aqueous solution showed self-aggregations of extended chains to align in parallel structure below 35 °C, which resulted in nanofiber composite hydrogels of mechanically high strength.	[94]
Chemically responsive SMHs	Alginate and hyaluronic acid Extruded technology	Authors used hybrid polymer, such as methacrylated alginate (AA-MA) and methacrylated hyaluronic acid (HA-MA). A hollow, self-folding tube was constructed by extruding polymer with or without cell on glass or polystyrene plate and subsequent polymerization in green light. The design print was immerged in water, PBS, and cell culture media for spontaneous self-folding and tube formation. The hydrogel (AA-MA) was tubular in water and became Ca^+2^-induced cross-linked hydrogel in CaCl_2_ solution to prevent swelling and formed a stiff film (unfolded). The unfolded hydrogel can be refolded by placing the AA-MA hydrogel in EDTA solution (EDTA removed Ca^+2^ ion).	[7]
	Acrylic acid	Hu et al. fabricated acrylic acid hydrogel for different swelling behaviors in alkaline and acid solution. The carboxylic group of side chain was highly ionized by protonation at pH ˃ 9 and vice versa. In the former case, there was electrostatic repulsion-based volume expansion, whereas volume shrinkage was observed in acidic medium due to reduced protonation. Two phases (cubic and circular plate). The relative expansion ratio (RER) was the same (0.43), suggesting that expansion was related to the material, not the shape. The RER values were 0.52 and 0.53 for the circular and the cubic plate at pH ˃ 9.0, respectively, whereas these values were 0.066 and 0.072 at pH ˂ 9.0, respectively.	[95]
	Acrylic acid monomer, PEG-diacrylate (as cross-linker) 2,4,6-trimethylbenzoyl-diphenylphosphine oxide (TPO)	Larush and his colleagues used water, acrylic acid, PEG-diacrylate, and TPO in nanoparticles (as photo-initiator) to design a cross-link polymerizable ink. The TPO facilitated the printing of the object in water with high resolution in SLA technology (stereolithography). The study aimed to deliver the loaded drug in alkaline intestine not in acidic stomach.	[96]
Magnetically responsive SMHs	Fe_3_O_4_ nanoparticle and algae-derived alginate ionic hydrogel	Authors developed 4D-printed soft aquatic actuator with magnetic NPs encapsulated in algal hydrogel with the movement speed of 0–4 mm/s or less in response to the magnetic flux of 400–700 G.	[97]
	Fe_3_O_4_ nanoparticle, laponite, and PDMAAm (poly-(*N,N*′-dimethyl acrylamide))	Lee et al. used magnetic NP-based hydrogel for culture cells. They reported good mechanical strength (toughness strength = 4198 KJ m^−3^ and Young’s modulus of 0.035 MPa) on addition of laponite. Culture growth was convincing and biocompatible as compared to conventional gel (PDMAAm hydrogel).	[98]
Photoresponsive SMHs	Alginate and polydopamine (PDA) scaffolds	This scaffold was reported to be folded under dehydration using near-IR radiation. PDA is an excellent biocompatible and photothermal scaffold capable of absorbing heat to accelerate the dehydration process for shape morphing. The bending process of PDA-based hydrogel was controlled by the power and exposure time to IR. The bending time was remarkably decreased (from 800 s to 160 s) by increasing the laser power (from 0.3 to 1.5 Wcm^−3^).	[99]
	Pluronic F127 diacrylate macromer (F127DA) Graphene oxide (GO)	The use of F127DA-based hydrogels for drug delivery in response to near-IR radiated exposure. Addition of GO allowed absorption of IR radiation for light-responsive changes and studied (a) the original shape at 37 °C, (b) the original shape after 5 min of IR radiation, and (c) the temporary shape after 5 min of IR radiation. The difference in drug release was due to the different surface transformed after IR radiation, such as temporary shape exhibited lower release rate due to twisted surface.	[100]
Electroresponsive SMHs	Maleic anhydride PVA Poly[(sodium maleate-co-sodium acrylate)]	Authors prepared PVA/poly(MSA-SAA) hydrogel by repeated frost-defrost process due to potential high charge density and sensitivity to electrical stimuli. The bending angle was dependent on several factors, such as hydrogel composition and type, concentration of electrolyte (NaCl) solution, and supplied electric voltage. Hydrogels demonstrated excellent recovery in on-off electric stimuli.	[101]
Dual stimuli responsive	Perylene bisimide-functionalized hyperbranched polyethylenimine (PBI-HPEI) Graphene oxide-PNIPAm (GO-PNIPAm)	Authors designed hydrogel responsive to pH and temperature. Graphene oxide-PNIPAm (GO-PNIPAm) layer was used to block the excitation light (532 nm green laser light), and no fluorescence was observed at initial stage. When the temperature reached a certain degree, the thermal responsive actuator of the GO-PNIPAm layer opened (unwrapped) the flower-like device. The fluorescence intensity was controlled by changing the pH. Intensity was inversely related with pH	[102]
	PAAm (polyacrylamide) and PAAc (poly(acrylic acid))	Dual-responsive actuator hydrogel wherein PAAm as a nonthermally activated polymer exhibited UCST (upper critical solution temperature) property on addition of PAAc. The bending angle of actuation was influenced by the ions (Cl^−1^, SCN^−1^, SO_4_^−2^, urea) present in electrolyte solution (5–60 °C).	[103]

**Table 5:** A summary of comparison between 3D- and 4D-printing techniques [112,113].

3D Technique	4D Technique
It contains the commands to print layers of material successively.	The technique adds a precise geometric code to the process as per desired shape.
**Materials**: Commonly used materials are clay, ceramics, metals, thermoplastics, printing paper, food-based materials, synthetic or natural polymers, nanomaterial, and biomaterials.	**Materials**: Smart shape-morphing materials/multimaterials, responsive to stimuli, and advanced materials
**Design concept**: It prints by drawing or scanning using the 3D digital object.	**Design concept**: It prints 3D digital object with deformation feature.
**Transformation**: 3D-printed objects cannot transform themselves over time.	Transformation: 4D-printed objects can transform themselves over time in one-way, two-way, and multiway.
**Printer**: Three-dimensional printer is required.	**Printer**: Four-dimensional printer is required.
**Processing**: Three-dimensional-printing technique is additive manufacturing obtained by adding rather than subtracting or shaping material by cold and hot techniques.	**Processing**: Four-dimensional-printing technique uses a process that produces a smart 3D construct/scaffold/object using shape-morphing materials under certain stimuli.
**Dynamicity**: The object created using a 3D printer can be static or flexible depending on the nature of materials.	**Dynamicity**: The smart object created using a 4D printer can undergo a real transformation (reversible) by itself under stimuli.
**Properties**: Materials have no self-assembling, self-adaptability, or self-healing properties.	**Properties**: Smart materials have self-assembling, self-adaptability, and self-healing properties.
**Strength**: Low cost, high efficiency process, customized model, positive market trend, high product quality	**Strength**: Efficient materials and process, positive market trend, multicolor material printing, smart materials, R&D on multimaterials printing
**Weakness**: Expensive equipment, production time high, quality differs using different printers, limited material selection, postprocessing may be needed, product size issue	**Weakness**: New technique, limited and expensive smart materials, expensive equipment and ink, relevant accuracy class, complex shapes, specialized operator needed
**Opportunity**: Customized design, recycling, smart materials, new equipment invention, develop potential materials	**Opportunity**: Remote operation, extreme environment, implant in medical, smart materials, 5D, and multiple materials
**Threat**: Machine compatibility, software upgradation, environmental impact, intellectual property right (IPR), market competition	**Threat**: Machine and software compatibility, software upgradation, public safety, IPR, market competition, environmental impact, maintenance
**Market output**: Medium	**Market output**: Medium-high
**Rheology**: Three-dimensional viscous hydrogel ink is nonapplicable due to rheological issue.	**Rheology**: Four-dimensional shear-shinning hydrogel ink is applicable for biomedical application.
**SWOT** rating for strength, weakness, opportunity, and threat is 9, 7, 8, and 7, respectively; overall rating = 31.	**SWOT** rating for strength, weakness, opportunity, and threat is 7, 6, 9, and 7, respectively; overall rating = 29.

Note: SWOT: Strength, weakness, opportunity, threat; R&D: Research and development.

## Data Availability

The data presented in this study are available on request from the corresponding author.
